# Gingival telangiectases due to dermatomyositis

**DOI:** 10.1002/jgf2.365

**Published:** 2020-08-27

**Authors:** Kiyoshi Shikino, Nao Hanazawa, Kazutaka Noda, Masatomi Ikusaka

**Affiliations:** ^1^ Department of General Medicine Chiba University Hospital Chiba Japan

**Keywords:** dermatomyositis, gingivitis, oral lesion, periungual erythema

## Abstract

We report the case of a 36‐year‐old woman who was referred to our hospital with a 10‐week history of intractable gingivitis. Gingival telangiectases may represent the sign of dermatomyositis. Early identification is essential for diagnosis and immediate treatment.

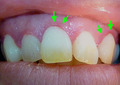

A 36‐year‐old woman presented with a 10‐week history of gingival pain. Physical examination revealed diffuse gingival telangiectases (Figure 1), Gottron’s papules (Figure 2), periungual erythema, and erythematous palmar macules, which had developed only 3 weeks prior to her visit. One week after visiting our hospital, symmetric weakness of the proximal muscles developed with increased muscle enzyme levels (creatine kinase was 592 U/L, aspartate aminotransferase was 97 U/L, and lactate dehydrogenase was 393 U/L). C‐reactive protein was 0.5 mg/dL, and ESR was 9/hr. Aminoacyl‐tRNA synthetase autoantibodies were negative. Electromyography showed evidence of increased insertional irritability with spontaneous fibrillation, short‐duration, polyphasic motor unit potential, and repetitive discharge. Based on these findings, the patient was diagnosed with dermatomyositis. After starting prednisolone and tacrolimus, all of the patient’s symptoms, including gingival telangiectases, improved.

**FIGURE 1:**
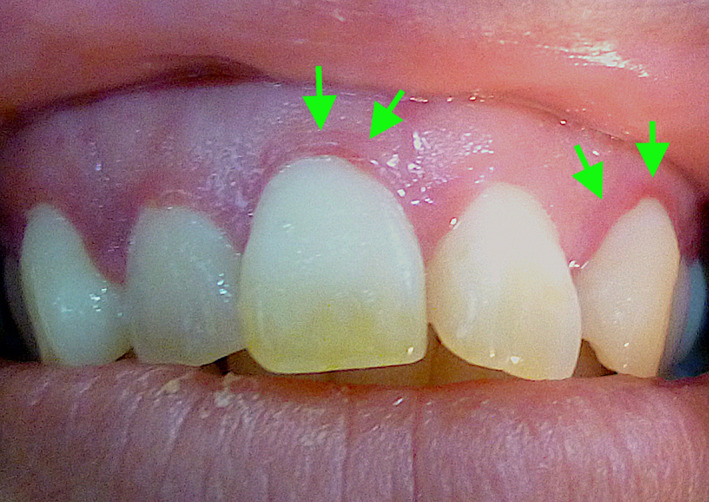
Gingival telangiectases (arrows)

**FIGURE 2:**
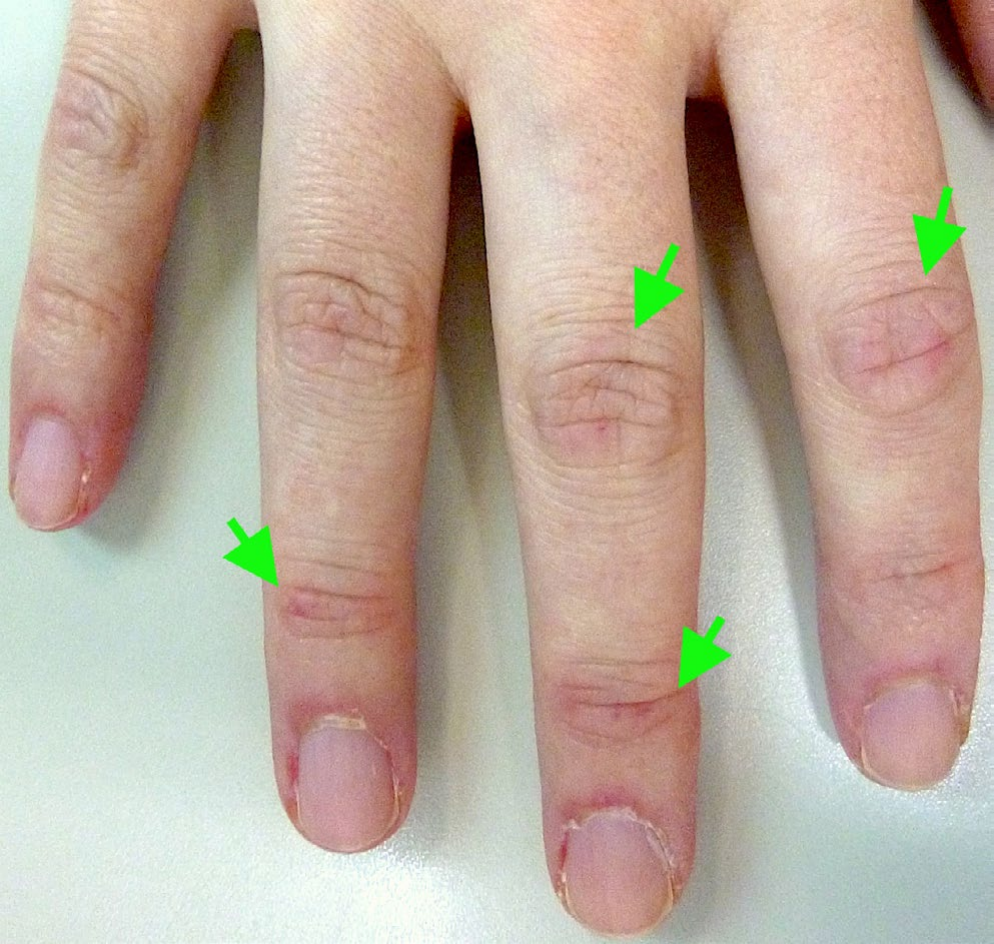
Gottron’s papules (arrows)

Gingival telangiectases may be significant in identifying subsets of dermatomyositis.^1^ Capillary abnormalities in the gingiva have been previously described in five patients with dermatomyositis^2^, the mechanism of which is speculated to be similar to that of periungual erythema.^1^, ^2^, ^3^ There is also one previous report of a patient with multiple oral lesions, including gingivitis, as the initial symptom of dermatomyositis.^3^ Thus, gingival telangiectases may represent the sign of dermatomyositis. Early identification is essential for diagnosis and immediate treatment.

## CONFLICT OF INTEREST

The authors have stated explicitly that there are no conflicts of interest in connection with this article.

## AUTHOR CONTRIBUTIONS

All authors had access to the data and a role in writing the manuscript.

## INFORMED CONSENT

We have obtained the consent of the patient for publication.
